# Deconstructing Dizziness

**DOI:** 10.3389/fneur.2021.664107

**Published:** 2021-04-29

**Authors:** Diego Kaski, Daniel Herron, Parashkev Nachev

**Affiliations:** ^1^Department of Clinical and Motor Neurosciences, Institute of Neurology, University College London, London, United Kingdom; ^2^National Institute for Health Research University College London Hospitals Biomedical Research Centre, London, United Kingdom; ^3^Department of Brain Repair & Rehabilitation, Institute of Neurology, University College London, London, United Kingdom

**Keywords:** dizziness, vestibular, ground truth, sensory integration, multimodal

## Introduction

Few of us are strangers to dizziness. As with pain, to equivocate about being dizzy is to cast doubt on one's mastery of the language, not to express uncertainty about the experience. The practiced ease of first-person use, however, conceals great difficulty in defining the criteria for correct ascription from which a clear picture of the symptom can be derived, and on which close scientific investigation is inevitably premised.

By “dizzy” a patient may mean any one—or combination—of vertigo, oscillopsia, light-headedness, spatial disorientation, or unsteadiness (1). Though primarily perceptual, the experience is commonly coupled with an incapacity to act or move appropriately, creating a complex sensorimotor blend. Superimposed is an emotional reaction to the profound dysfunction the patient takes the symptoms to imply. How do we decompose so polymorphous a phenomenon; what dependencies can we establish between its components; and how do we relate them to the underlying neural substrate, in health and disease? These are the questions we wish to answer: we shall see we must be wary of the answers they immediately prompt in us, for intuition is here misleading.

## The Nature of Dizziness

Prima facie, dizziness has perceptual, motor, and emotional components. Let us take each in turn.

### Perceptual

It is natural to think of dizziness as an abnormal sensation of body movement in space. If so, it ought to be dependent on the integrity of a perceptual power. The blind cannot be dazzled by headlights; the deaf startled by a bang; the anosmic overwhelmed by perfume. And if the deficit is congenital, then these experiences are *logically* proscribed, for there is no framework within which their expression could have been learnt (2). But what sensory modality must a patient lack to be incapable of dizziness?

It cannot be the vestibular sense, for the kind of illusory head motion commonly associated with vertigo falls within the repertoire of normal motion *as registered by the vestibular system alone*. Moreover, inactivation—partial or complete—of the vestibular system does not attenuate or prevent dizziness (although patients may not experience rotational vertigo) but amplifies or causes it (3, 4). It cannot be vision either, for the same reasons: the visual correlates of dizziness are typically replicable without it, and though an image, especially a moving image, may trigger vertigo, closing one's eyes does not universally abolish it (5). The experiential volume of proprioception is arguably too weak to carry so vivid an experience, but the same arguments apply in any event.

So the perceptual aspect of dizziness is not explicable by any *single* perceptual modality. Rather it requires the interaction of at least *two*, as classically illustrated by the caloric reflex test. Here artificial stimulation of the vestibular apparatus using water at varying temperatures creates a discrepancy between artificially stimulated vestibular and intact visual signals, generating nystagmus accompanied by vertigo (6). Removing visual input by closing one's eyes attenuates the experience but does not abolish it, for proprioceptive signals remain at odds.

Examples of other multi-modal combinations are easy to give. But what is the nature of the critical cross-modal interaction? A cross-modal comparison can never be direct, for the signals of each modality are definitionally different. But we can compare the circumstances under which a given perceptual signal is obtained: here typically a coherent pattern of motion of the eyes and head. Dizziness generally arises where the associated circumstances—real or merely predicted—are discordant. Crucially, it is the mere *presence* of discordance—not its direction, quality, or magnitude—that evokes the experience (7). To the extent to which dizziness is perceptual it is *meta-perceptual*, superordinate on the sensory modalities whose discordance it registers. This places it in a unique experiential category: an indicator of the cross-modal coherence referenced to the body. We cannot easily construe it on the model of simple sensations, for its perceptual aspect is *sui generis*.

### Motor

Any experience involving the perception of movement is bound to exhibit a motor aspect (8). Though affordance is widely assumed to be specific to the spatial properties of objects of action (9), there are no grounds for believing it must be so limited. Indeed, if action is to be responsive to the spatiotemporal continuity of the environment, affordance must extend both to the subject, and across time (10). If I erroneously perceive myself to be falling backwards, then when I make no corresponding motor response it is only because I have deliberately *suppressed* it in the realization the perception is illusory. Here the motor system is naturally activated downstream of an afferent signal—the movement, or suppressed movement is reactive—but its contribution to the experience need not be secondary.

Nowhere are action and perception more entangled than in the visual system. The primary objective of eye movements is to maintain a tight coherence of gaze and environmental salience over time (5). The global, background shift implied by a perception of self-motion—illusory or real—cannot but activate the oculomotor system, which must act automatically to stabilize an image that would otherwise become uninterpretable (11). Indeed, it is on the oculomotor system that the cross-modal comparison between the visual and the vestibular depends. The vestibular system needs to integrate multimodal signals to determine where the head is in space, and to where our gaze should be directed.

In short, collateral motor phenomena—present or merely expected—accompanying the perception of motion create a motor aspect that is impossible to ignore, and whose contribution to the experience cannot be discounted merely for being subordinate to the perceptual.

### Emotional

Dizziness is characteristically accompanied by a visceral response far removed from its causal locus: nausea and vomiting. If a maladaptive instinctual reaction can be so prominent here, why could it not extend into the emotional realm, where rationality plausibly has a firmer purchase? The spatial disorientation often accompanying dizziness creates a sensory discordance that rightly generates instinctually-mediated distress (12). Again, that the emotional response is here reactive does not allow us to disentangle it from the rest of the experience, for its qualities may be peculiar to these circumstances. For example, a patient with benign paroxysmal positional vertigo [BPPV] usually not only has vertigo, but a consequent sense of loss of control. This creates secondary emotional symptoms—derealization and depersonalization—reflecting a radical redescription of the environment and the patient's interaction with it (13). Indeed, the secondary emotional disturbance may dominate the clinical landscape, resulting in a patient with dizziness receiving a primary diagnosis of anxiety (14, 15).

## Neural Consequences

**Figure 1:**
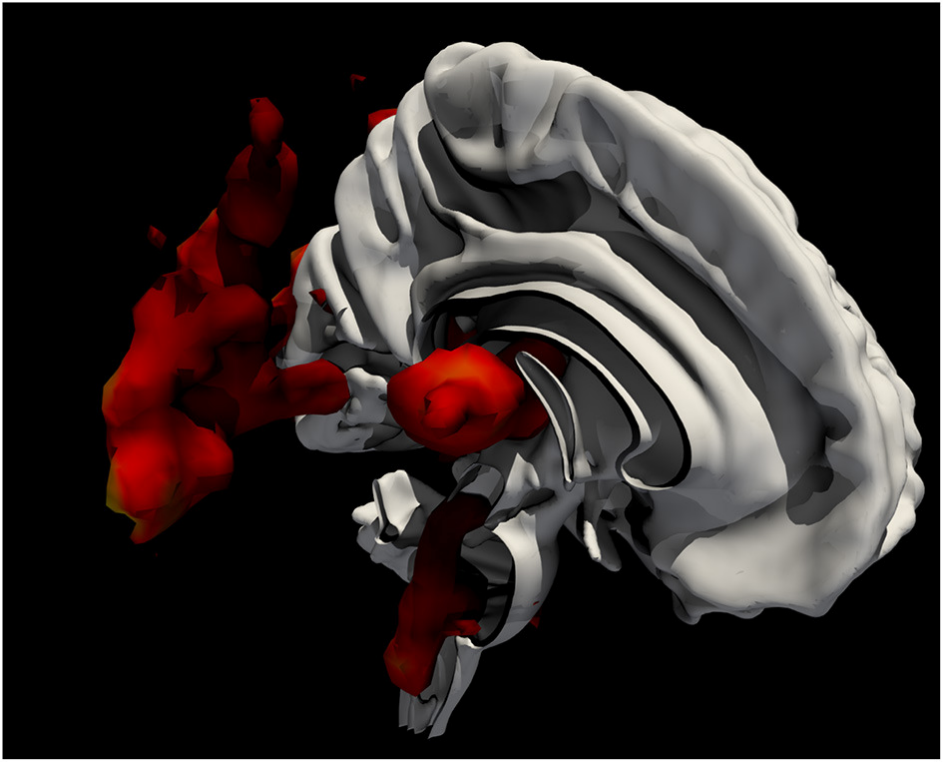
Ray-traced, contoured representation—overlaid on a thresholded white matter map—of meta-analytically derived functional imaging activations in research studies involving the keyword “vertigo,” generated from https://neuroquery.org/query?text=vertigo+.

We have seen that dizziness presents complex cross-modal perceptual, motor, and emotional aspects whose interdependence is not easy to disentangle, for their coincidence here is at least in part unique. Those attempting to identify its neural substrates cannot focus on a single perceptual modality and cannot easily set up a physiological contrast that sharply isolates one domain from another (Figure 1). Crucially, it makes little sense to seek a discrete substrate where a phenomenon is driven by discordance that may range widely across at least three sensory modalities, and whose motor and affective components may be expected to be commensurately diverse. Rather, it is the connective, hodological aspects of the underlying organization that are plausibly critical here, likely distributed across the brain (1, 16, 17). Since discordance in the realm of motion is inevitably time-bound, operating over fine temporal scales, so will be the neural phenomena that underly it. In short, without whole-brain, sharply-time resolved, high-resolution, multinodal graphical representations of the brain that no single investigational modality currently provides, the neural mechanisms behind dizziness will be hard to illuminate.

Here conceptually motivated, these observations are strikingly reflected in the relation between dizziness and pathological neural dysfunction. The special case of vestibular dysfunction aside, dizziness is often more likely to be evoked by mild—but diffuse—dysfunction than severe, focal dysfunction, and both marked symptomatology and objective measures of sensory integration may be present in the absence of visible neural damage, for example in closed traumatic brain injury (18, 19).

## Sensory Integration without an Integrator

It is tempting to take the notion of sensory discordance as implying the existence of a dedicated “sensory integrator” whose failure is its cause (20). The idea mirrors the supposed process of “binding” distinct sensory features into the spatiotemporal continuities that define objects. Let us consider why the idea is misguided, here as in the wider “binding” literature (21).

For an integrator to determine the concordance or discordance of two signals it must logically have a criterion for differentiating between the two possibilities: it needs a ground truth. But neural signals from two different modalities are definitionally different, and unless their correspondence is genetically encoded—hard to imagine given the constraint on genetic space—it can only be learnt from the intrinsic structure of multi-modal sensory experience, not any kind of label, for no such label is available. As stereotyped patterns of sensory correspondence emerge in the interaction of the organism with its environment, the structure of these patterns is determined by that interaction, becoming the ground truth from which discordance registers. We neither need—nor can have—an integrator, for structure emerges through self-supervision, analogously to artificial neural network autoencoders (22).

Note this implies a dynamic state open to revision, and sensitive to context, which is indeed what we observe. In the case of visual dependence (23)—overreliance on visual inputs that often causes a sense of dizziness and a fine example of visuo-vestibular interaction. Certain individuals rely strongly on visual input such that when they are inside a tilting room their perception of verticality is significantly biased in the direction of the visual tilt (11, 24). The degree of visual dependence is itself dependent upon the reliability of the sensory signals involved in spatial orientation and postural control. Thus, during the microgravity phase of parabolic flight, where inertial vestibulo-proprioceptive cues are reduced, the weight given to vision is potentiated (25). The association between visual dependence and psychological traits (26, 27) was shown to be of clinical relevance in patients with acute unilateral vestibular failure whereby an interaction between psychological, vestibular and visual dependence shapes recovery after vestibular neuritis (28).

The ground truth must change contextually–a coherent integration of all the visual, vestibular and proprioceptive senses for a given environment (external and endogenous) and emotional state. In cases of BPPV (where mis-placed crystals erroneously induce vestibular nerve firing), the patient's vestibular system fails transiently, distorting a sensory signal a brain ordinarily expects to cohere with the rest of the sensorium, and because the change is transient, compensatory adaptation cannot easily take place (29).

The natural flexibility of integration—over context and time—reflects not a weakness of the system but the only licit means by which integration can be achieved. It explains both the phenomenology of pathological insults and the capacity of the system to adapt in response. Here, as elsewhere in the brain, flexibility is not primarily a reflection of neural resilience, but of the fundamental mode of operation.

## Conclusions

Reflection on the conceptual nature of dizziness should cause us to reconsider our approach to studying its neural substrates. Such connective analysis can guide empirical investigation by defining the logical bounds of empirical possibility, ensuring the hypotheses we generate and test lie within it. For a hypothesis that makes no sense—such as that dizziness is a manifestation of vestibular failure or the dysfunction of a sensory integrator—can be neither true nor false, only senseless. The utility is here not merely intellectual-hygienic, for careful exploration of the horizon possibility draws attention to hypotheses—not just about physiology but also about treatment—mistaken intuition may have previously obscured. For example, independence from the precise form of sensory discordance that dizziness symptomatically registers suggests the use of vestibular measures in the assessment of distributed neural dysfunction, whether symptomatic or not. In short, armchair reflection can both save us from fruitless empirical adventure and cast light on new avenues with great clinical potential.

## Author Contributions

DK contributed to the conceptualization, manuscript compilation, and final approval. DH was responsible for the conceptualization of the manuscript and content. PN contributed to the conceptualization, compiled the manuscript, and approved final manuscript. All authors contributed to the article and approved the submitted version.

## Conflict of Interest

The authors declare that the research was conducted in the absence of any commercial or financial relationships that could be construed as a potential conflict of interest.
